# Biochar Amendment Modifies Expression of Soybean and *Rhizoctonia solani* Genes Leading to Increased Severity of Rhizoctonia Foliar Blight

**DOI:** 10.3389/fpls.2017.00221

**Published:** 2017-02-21

**Authors:** Tanya Copley, Stéphane Bayen, Suha Jabaji

**Affiliations:** ^1^Plant Science Department, McGill UniversityMontreal, QC, Canada; ^2^Department of Food Science and Agricultural Chemistry, McGill UniversityMontreal, QC, Canada

**Keywords:** biochar, rhizoctonia foliar blight, reduced immunity, gene expression, primary and secondary metabolism, soybean, phytohormones

## Abstract

Application of biochar, a pyrolyzed biomass from organic sources, to agricultural soils is considered a promising strategy to sustain soil fertility leading to increased plant productivity. It is also known that applications of biochar to soilless potting substrates and to soil increases resistance of plants against diseases, but also bear the potential to have inconsistent and contradictory results depending on the type of biochar feedstock and application rate. The following study examined the effect of biochar produced from maple bark on soybean resistance against Rhizoctonia foliar blight (RFB) disease caused by *Rhizoctonia solani*, and examined the underlying molecular responses of both soybean and *R. solani* during interaction with biochar application. Soybean plants were grown in the presence of 1, 3, or 5% (w/w) or absence of maple bark biochar for 2 weeks, and leaves were infected with *R. solani* AG1-IA. At lower concentrations (1 and 3%), biochar was ineffective against RFB, however at the 5% amendment rate, biochar was conducive to RFB with a significant increase in disease severity. For the first time, soybean and *R. solani* responsive genes were monitored during the development of RFB on detached leaves of plants grown in the absence and presence of 5% biochar at 0, 6, 12, and 24 h post-inoculation (h.p.i.). Generally, large decreases in soybean transcript abundances of genes associated with primary metabolism such as glycolysis, tricarboxylic acid (TCA) cycle, starch, amino acid and glutathione metabolism together with genes associated with plant defense and immunity such as salicylic acid (SA) and jasmonic acid pathways were observed after exposure of soybean to high concentration of biochar. Such genes are critical for plant protection against biotic and abiotic stresses. The general down-regulation of soybean genes and changes in SA hormonal balance were tightly linked with an increased susceptibility to RFB. In conjunction, *R. solani* genes associated with carbohydrate metabolism were up-regulated, while genes involved in redox reactions and detoxification had varying effects. In conclusion, this study presents strong evidence that maple bark biochar increased susceptibility of soybean to a foliar disease. This condition is partly mediated by the down-regulation of soybean genes leading to reduced immunity and also affecting *R. solani* gene expression.

## Introduction

Biochar, a pyrolyzed biomass prepared from a variety of organic sources, has been a subject of an increasing number of articles, mainly fueled by its positive effects on plant growth and the potential to increase crop yields (Lehmann and Joseph, 2009); however, negligible to adverse effects are also commonly reported in biochar experiments (Spokas and Reicosky, 2009; Jeffery et al., 2011; Spokas et al., 2011). It is not a surprise to have contradictory results since biochars prepared from different biomass types differ in their production parameters, and physicochemical and biological properties (Lehmann and Joseph, 2009). These differences have been reported to alter biochar subsequent effects on soil quality and crop productivity (Spokas et al., 2011; Mukome et al., 2013). Applications of biochar to soilless potting substrates and to soil have been reported to increase resistance of plants against disease, but also bear the potential to have inconsistent and contradictory results depending on the type of biochar feedstock, application rate and the pathosystem.

Generally, biochar amendments have been reported to reduce the severity of fungal foliar diseases such as powdery mildew, anthracnose and gray mold (Elad et al., 2010; Meller Harel et al., 2012; Mehari et al., 2015) with evidence that disease severity is biochar dose-dependant. Results from studies examining biochar amendments on fungal root diseases, however, are inconsistent: enahnced disease incidence of *Fuasrium oxysporum* f. sp. *lycopersici* in tomato was reported in biochar amended substrate (Akhter et al., 2015), while Fusarium crown and root rot in asparagus was suppressed at low concentrations of biochar, but increased at greater biochar concentrations (Elmer and Pignatello, 2011). Biochar amendment had no effect on suppression of Phytopthora blight in sweet pepper (Shoaf et al., 2016), results similar to those observed with *Pythium ultimum* in lettuce, sweet pepper and herbs, albeit higher *Pythium* root colonization rates were observed (Gravel et al., 2013).

Despite the increasing research on this issue, there is a general lack of understanding of how biochar amendments affect factors related to rhizospheric microbes, host susceptibility, and pathogen virulence. It has been suggested that biochar type and dose-rate may affect host susceptibility and pathogen virulence (Graber et al., 2010; Jaiswal et al., 2015). In accordance with this notion, we recently examined the effect of biochar on *Rhizoctonia solani* Kühn anastomosis group 4 (AG4) as a root pathogen, and showed that at low concentrations, biochar reduced or had no effect on damping-off incidence and severity in soybean (*Glycine max* (L.) Merr.) (Copley et al., 2015b). However, at higher concentrations, biochar was conducive to disease development in soybean, as well as in a variety of plant species (Copley et al., 2015b).

One of the factors that contributed to increased damping-off incidence caused by *R. solani* is likely linked to the ability of the pathogen to metabolize organic compounds present in maple bark biochar such as oxalic acid, benzoic acid, glycerol, and ricinoleic acid leading to an increase in its growth rate, which might promote virulence (Copley et al., 2015b). These metabolites are known for their stimulatory effect on fungal growth and can be metabolized by several fungi (Sunesson et al., 1995; Douds et al., 1996; Fries et al., 1997; Matsuzaki et al., 2008). Indeed, significant linear extension and increased growth rates of *R. solani* were demonstrated when the pathogen was grown on water agar amended with maple bark biochar as a carbon source relative to non-amended plates. This was positively correlated with increases in sugar alcohol concentrations in hyphal cells of *R. solani* grown on greater biochar rates, possibly leading to increased pathogenicity and virulence (Copley et al., 2015b). Despite the importance of understanding how biochar may affect pathogen virulence and metabolism, no additional studies to date have addressed the direct effect of biochar on pathogen growth and virulence.

To date, limited studies have examined the underlying plant molecular responses to biochar application. Global Arabidopsis transcriptome fluctuations in response to high biochar amendment rates (100 tons ha^−1^), but not when subjected to pathogen attack, showed that many of the genes related to plant immunity and defense were down-regulated (Viger et al., 2014). These results point to a complex interaction between biochar and plants, suggesting that more studies are required to determine if changes in gene expression result in reduced plant immunity when plants are subjected to pathogen attack.

Taken together, we set out to determine whether increasing amendment rates of maple bark biochar would produce similar results to what we previously reported on a soilborne pathogen, when soybean is subjected to a foliar pathogen belonging to the same taxonomic group, and whether plant and pathogen responsive genes are affected. Rhizoctonia foliar blight (RFB) of soybean, caused by *R. solani* AG1-IA can result in yield losses up to 60% (Fenille et al., 2002; Stetina et al., 2006) in Brazil and the southern states of the USA. Analysis of RNA sequencing of soybean-RFB interactions showed that plant genes involved in photosynthesis metabolic pathway were down-regulated with concomitant up-regulation of genes associated with amino acid and carbohydrate pathways and the tricarboxylic acid (TCA) cycle, presumably to provide energy, and carbon and nitrogen sources for secondary metabolism and defensive compounds (Copley et al., 2015a). From the pathogen side, several genes associated with fungal primary metabolism were differentially expressed (Copley, unpublished data). How biochar soil amendment may affect soybean and RFB pathogen-associated genes during their interaction merits investigation, considering that the location of biochar is spatially separate from the site of infection which would indicate there was no direct effect toward both the plant and the causal agent, and points to an indirect mechanism related to plant and pathogen responses.

Here we report on whether the application of biochar to potting mix (i) affected RFB disease severity and caused changes in soybean gene abundance and plant hormones, and (ii) whether the expression of plant and pathogen genes were altered during the interaction between soybean and *R. solani* leading to reduced immunity.

## Materials and methods

### Biochar production and physiochemical properties

Biochar, supplied by Awards Rubber and Plastic Industries Ltd. (Plessisville, Canada), was produced by pyrolysis of maple bark at 700°C for 4 h and used as an amendment. Biochar was ground and sieved to obtain particle sizes ≤ 1 mm. Detailed physical, elemental and biochemical characterization of biochar powder can be found in Tables 2 and 3 in Copley et al. (2015b)

### Pathogen inoculum preparation

A virulent pathogenic strain of *Rhizoctonia solani*, isolate ROS-2A4, belonging to anastomosis group AG1-IA was provided by Dr. Paolo Ceresini, University of São Paulo State (UNESP), Brazil. The isolate was revived from stock cultures maintained at −80°C by placing a hyphal plug on fresh potato dextrose agar (PDA) for 1 week at 24°C in the dark. Cultures were then subcultured to fresh PDA containing sterile millet seeds and the culture allowed to fully colonize the millet seeds for 2 weeks at 24°C in the dark. Colonized millet seeds were used as an inoculum source for soybean infection.

### Plant inoculation and disease assessment

#### Experiment 1

To examine the effect of different concentrations of biochar on Rhizoctonia foliar blight (RFB) severity, soybean (*Glycine max*) cultivar Williams 82 seed were surface sterilized in 30% hydrogen peroxide for 7 min followed by 5 rinses in sterile water. Seeds were then imbibed on damp sterile filter paper for 48 h until the root radicle emerged from the seed coat. Uniformly pre-germinated seeds were planted one per pot in 60 mL pots containing AgroMix G10 (Fafard Ltd., St. Bonaventure, Canada) and sand (1:1 v/v) amended with 0, 1%, 3, or 5% (w/w) biochar (equivalent to approximately 0, 25, 75, and 125 tons ha^−1^, respectively) by mixing the biochar within the potting substrate prior to planting. These concentrations were similar to those used in other studies examining the effects of biochar on plants grown in soilless potting mix. Plants were arranged in a complete randomized design (CRD) in a growth cabinet with 12/12 h of day/night, 25/23°C day/night temperatures, 210 photons μm^−2^ s^−1^, and humidity maintained at 65% throughout the entire experiment. Two-weeks post-planting, at the unifoliate stage, leaf chlorophyll content of fully expanded unifoliate leaves was quantified using a SPAD 502 meter (Konica Minolta Optics, Inc., New Jersey, U.S.A.) by averaging the reads of 10 readings per leaf. Unifoliate leaves were immediately detached from the seedlings grown in the absence of biochar (0%) and in biochar-amended potting mix, placed on sterile moistened filter paper in Pyrex® dishes (25 × 15 cm), and arranged in a complete randomized block design (CRBD). Unifoliate leaves of each seedling were inoculated with a millet seed fully colonized with *R. solani* by placing it in the middle of the leaf. The Pyrex dishes were wrapped in saran wrap and placed in a growth cabinet under the conditions described above. Disease severity was recorded 24 h post-inoculation (h.p.i.) and photos for disease assessment were taken using Image J software version 1.49 (Abràmoff et al., 2004). RFB disease assessment was performed by bleaching leaves using 3:1 chloroform:methanol (v/v) until all chlorophyll was removed for better visualization of the necrotic area caused by *R. solani*. The level of necrosis was determined by calculating the amount of yellow-brown (necrotic) pixels compared to the entire leaf area using Image J software and expressed as percent leaf area infected (Abràmoff et al., 2004; Li et al., 2015). Six leaves, from six different plants, per treatment were analyzed in each trial for a total of two trials and 12 leaves per treatment.

#### Experiment 2

Another set of experiments was conducted to study the development of disease prior to the onset of symptoms and to examine the effect of biochar on expression of soybean and *R. solani* genes. Detached leaves from 2-week-old plants (unifoliate stage) grown in the absence of biochar (0%) and in 5% biochar amended potting mix, the concentration that had the strongest effect on RFB disease severity, were inoculated with *R. solani* colonized millet seed under the same conditions as described above and experimentally arranged in CRD. Disease progression and severity were recorded 6, 12, and 24 h.p.i. by measuring necrotic regions as percent leaf infected with Image J software as previously described. At early stages of infection (i.e., 6 and 12 h.p.i) where no necrosis had occurred, hyphal expansion was measured instead by staining the hyphae with lactophenol blue post-leaf-bleaching. The level of hyphal expansion was determined by calculating the amount of blue (hyphae) pixels compared to the entire leaf area using Image J software and expressed as percent leaf area infected (Abràmoff et al., 2004; Li et al., 2015). Six leaves from six different plants per treatment were analyzed in each trial for a total of two trials, and 12 leaves per treatment per time point.

For the gene expression study, leaf areas containing the *R. solani* hyphae plus an additional 0.5 cm beyond the hyphal limit were harvested at 6, 12, and 24 h.p.i from leaves of seedlings grown in the absence and presence of biochar, and frozen in liquid nitrogen. Six excisions were pooled together for one biological replicate, and a total of three replicates per time point per treatment were analyzed using qRT-PCR and HPLC-MS for gene expression and hormone analyses, respectively. In parallel, leaves from plants grown in 0 or 5% biochar but not subjected to infection (i.e., 0 h.p.i.) were also collected from 2-week old seedlings to determine the effect of biochar on soybean gene expression. Detached leaves from all treatments were flash frozen in liquid nitrogen.

### RNA extraction, cDNA synthesis, and qRT-PCR

Total RNA from leaves of all treatments and time points was extracted from 100 mg of infected leaf tissue using the RNeasy plant mini kit (Qiagen, Toronto, Canada) following the manufacturer's protocols. RNA quality was confirmed on a denaturing formaldehyde agarose gel (2%) and quantified using a NanoDrop. cDNA was synthesized using the iScript Advanced cDNA Synthesis for RT-qPCR (Bio-Rad Laboratories, Ltd., Mississauga, Canada) using 2 μg of total RNA from all time points, including 0 h.p.i.

To determine if exposure to biochar has an effect on the transcript abundance of soybean and *Rhizoctonia* responsive genes following *R. solani* infection, 14 soybean genes commonly associated with primary metabolism (i.e., involved in glycolysis, the TCA cycle, starch metabolism, amino acid and glutathione metabolism) together with 5 genes associated with secondary metabolism and plant defense, and 13 *R. solani* genes (Table 1) were normalized against plant and fungal housekeeping genes, respectively, and quantified relative to the control treatments by qRT-PCR. Briefly, each 20 μL reaction contained 1X SsoAdvanced Universal SYBR Green Supermix (Bio-Rad Laboratories Ltd.), 0.175–0.25 μM each primer (Table 1), and 600 ng cDNA for soybean transcript quantification, or 900 ng for *R. solani* transcript quantification. The thermocycling profile used an initial denaturation at 95°C for 3 min, followed by 35 or 40 cycles of denaturation at 95°C for 30 s, annealing for 30 s at the appropriate primer temperature and extension at 72°C for 40 s, followed by a dissociation curve analysis. Transcript abundance was analyzed using the method of Zhao and Fernald (2005) with normalization over the housekeeping gene encoding a hypothetical protein unknown (*UKN2*) for soybean transcripts (Libault et al., 2008) or *R. solani* histone 3 (ELU43810) for *R. solani* transcripts.

**Table 1:** **qRT-PCR primer sequences and thermocycling conditions**.

**Gene locus**	**Annotation**	**Primer name**	**Primer set (5′–3′)**	**Product size (bp)**	**Annealing temp. (°C)**	**Final primer concentration (μM)**
**SOYBEAN GENES^#^**						
GLYMA19G36620	Phenylalanine ammonia lyase 1 (*PAL*)	PAL1-F	GTCCAGTACTAAGGGAAGTGATCC	216	54	0.15
		PAL1-R	ACTCCTTCTCGGGCAGACTC			
GLYMA03G12240	Glutamate-5-kinase (*G5K*)	G5K-F	ACTCTTGCAAAATGGCCACA	160	54	0.175
		G5K-R	ACGCTTCACTTTGGTGACAA			
GLYMA10G33650	Glutathione-S-transferase (*GST*)	GST-F	GATGACATGTTTTCTGCAGTTATTG	174	54	0.2
		GST-R	CCCAAAAGCTATGTCCATAATGT			
GLYMA08G20230	Lipoxygenase 10 (*LOX10*)	LOX10-F	ATGCAAAAATGTACAAAAACACTCGTA	188	54	0.2
		LOX10-R	GGGTGTTCCCAAATCATTGT			
GLYMA08G45210	Alpha-glucan phosphorylase (*AGP*)	AGP-F	TTGAGCTGGAACAAGCTTACTAT	279	51	0.2
		AGP-R	GCCTACCAAGACCACCATTT			
GLYMA03G04990	Alanine-glyoxylate transaminase (*AGT*)	AGT-F	CTCAAAACTTCCCAGTGATCTC	156	51	0.25
		AGT-R	GCCATTGTCCCAGTTGCA			
GLYMA04G01950	Alpha-amylase (*AMY*)	AMY-F	GTCAGTGGAATCTGGTGGATAC	238	51	0.2
		AMY-R	CCAGGTAAGTCACATCCAACTTTA			
GLYMA02G39320	Asparagine synthetase (*ASN*)	ASN-F	GGTACAATCCTCCTTGGTTCTC	290	51	0.2
		ASN-R	GCCTAGATAGTCAGCAACTTCTT			
GLYMA15G10480	Beta-amylase (*BAMY*)	BAMY-F	AGTTCTTCTTGACCTGGTATTC	199	51	0.2
		BAMY-R	CGGTATCCGTCTCTATCATTAAG			
GLYMA05G04290	Beta-fructofuranosidase or invertase (*BFF*)	BFF-F	GAACGATCCCAATGGTCCTATG	248	51	0.2
		BFF-R	CGTTGGTGGAACCTGTGTATAA			
GLYMA12G05780	Beta-glucosidase (*BGLUC*)	BGLUC-F	GACTTCCAGTATGGATGGTTTAT	247	50	0.2
		BGLUC-R	CATCACGTACAAATGAGGAATTAG			
GLYMA01G24530	Delta 1-pyrroline-5-carboxylate synthase 2 (*DPSC2*)	DPSC2.1-F	AATTTCGTCAGCATCAAACC	259	50	0.2
		DPSC2.1-R	CCAATATGACTTCATACCCT			
GLYMA19G01200	Formate dehydrogenase (*FDH*)	FDH-F	ATGAACTCCTCAGAATCCTTGT	223	50	0.2
		FDH-R	GTATTCATCCTAAGTCTATCATAGTAC			
GLYMA17G13730	Malate synthase (*MLS*)	MLS-F	GAAGATCCAGTGGCTAACGAGGTAGC	204	58	0.2
		MLS-R	TTGCTCGGTGATGTTTGCCCCA			
GLYMA01G23790	Phosphenolpyruvate carboxykianse 1 (*PEPC*)	PEPC-F	GGTGAAAGATGAAGTTACTGAGAATG	251	51	0.25
		PEPC-R	CTTCGGTAGTTGGTCGAATG			
GLYMA09G02430	Non-expresser of PR protein 1 (*NPR1*)	NPR1-F	TTGAACCTGATTGCGATTATAG	147	54	0.2
		NPR1-R	ATTTCCCTTCTTTTTCTGATGA			
GLYMA05G06790	Pathogenesis-related protein 1 (*PR1*)	PR1-F	TGTTGCGTATGCTCAAGACT	195	54	0.2
		PR1-R	CACTTAGGTTACCGGTGCTT			
GLYMA02G04820	Pathogenesis-related protein 3 (*PR3*)	PR3-F	CGAGGACCAATCCAACTTAC	180	54	0.2
		PR3-R	AGTGATCACAT CATGGCTTG			
GLYMA18G48730	Ethylene-responsive element-binding protein 13 (*EREBP*)	EREBP1-F2	ATGTCTGAAACCCACCAAGC	181	54	0.15
		EREBP1-R2	CAAACTTTCCCCACGGTCTA			
GLYMA06G04180^*^	Hypothetical protein unknown 2 (*UKN2*)	UKN2-F	GCCTCTGGATACCTGCTCAAG	79	58	0.2
		UKN2-R	ACCTCCTCCTCAAACTCCTCTG			
***R. SOLANI*** **GENES^&^**						
ELU42665	Alpha-amylase (*RsAMY*)	RS_AMY_F	AAGCGAAGCTGGGAACAA	268	53	0.25
		RS_AMY_R	TAATATCCGCGAGTTGGTTGAC			
ELU39168	Thiamine biosynthesis (*RsTHI*)	RS_THI_F	TAATATCCGCGAGTTGGTTGA	136	52	0.25
		RS_THI_R	CCAAGCCTCTTCGAGTAGTTAG			
ELU42868	Beta-glucosidase (*RsBGLUC*)	RS_BGLUC_F	TGGTTCGCAGACCCTATTTA	201	52	0.25
		RS_BGLUC_R	GGTGTACTGGACGTTTCCTT			
ELU45264	Glutathione-S-transferase (*RsGST*)	RS_GST_F	GGATGCTAAGCTCGATGGATAC	190	52	0.2
		RS_GST_R	GGATGCTAAGCTCGATGGATAC			
ELU38450	Formate dehydrogenase (*RsFDH*)	RS_FDH_F	CCAAGAAGAACGAGCAGAAAT	158	52	0.25
		RS_FDH_R	TCCAGCTGCCTTGTACGACCT			
ELU42795	Cu/Zn superoxide dismutase (*RsSOD*)	RS_SOD_F	GCAAGATCACTGGCCTAACA	195	52	0.25
		RS_SOD_R	CAACTTTGGATTCGCCATTCG			
ELU41063	NADH oxidase (*RsNOX*)	RS_NOX_F	GTGTCGAATTTCAGGCGAAAG	244	52	0.25
		RS_NOX_R	CGGAATCCACCGGTAACATAA			
ELU43748	ABC transporter (*RsABC*)	RS_ABC_F	AGCATTTGGTGGTGATGTAGAA	224	52	0.25
		RS_ABC_R	CCAGGCTCTTTGCGATGTAATA			
ELU38592	Chitin deacetylase (*RsCDC*)	RS_CDC_F	GCATGACGTAGTCCTAAGAAGG	207	52	0.25
		RS_CDC_R	CTCGTTCCCGTCGCTATATTC			
ELU36963	Cytochrome P450 monooxygenase pc-12 (*RsP450*)	RS_P450_F	CAACCTATCGCAGTGGACTTT	117	52	0.25
		RS_P450_R	GTGAGGATAGGGAAGGGTAGAA			
ELU37123	Laccase precursor (*RsLAC*)	RS_LAC_F2	CCAAGGGCACGGCTATAAA	122	53	0.25
		RS_LAC_R	CATCTCGAACGATAGGGACAAG			
ELU40841	Pyridoxal-dependent decarboxylase (*RsPDX*)	RS_PDX_F	GAACAACCAAGCATTACTCGTG	80	53	0.25
		RS_PDX_R	GACCGGGACGTCAATGATATG			
ELU41358	Glycogen synthase (*RsGCS*)	RS_GCS_F	CTTATCCTGATGCCTTCGGTG	187	52	0.25
		RS_GCS_R	GGCCATACTTGACCCTTGTAATC			
ELU43810^*^	Histone 3 (*RsH3*)	RS_H3_F	CTTCCAATCATCGGCAGTCCTC	76	52	0.2
		RS_H3_R	ATTGGTATCTTCGAACAAAGACACGAG			

# *Genes selected for quantification based on Copley et al. (2015a)*.

* *Housekeeping genes used for normalization*.

& *Genes selected for quantification based on unpublished RNAseq analyses (Copley, unpublished data)*.

### Hormone analysis

To determine if biochar amendment affects soybean jasmonic acid (JA) and salicylic acid (SA) levels, the hormones were extracted from 100 mg of infected leaves of plants grown in the presence (5%) or absence of biochar at 0, 6, 12, and 24 h.p.i following the modified method of Pan et al. (2010). Briefly, hormones were extracted from 50 mg of ground leaf tissue suspended in isopropanol:water:hydrochloric acid (2:1:0.002 v/v) with shaking at 200 rpm at 4°C for 30 min followed by the addition of 2X volume dichloromethane and shaking at 200 rpm at 4°C for 30 min. Samples were then concentrated under vacuum centrifugation evaporation at 12°C using a Labconco CentriVap equipped with a cold trap (Labconco, Kansas City, MO), and re-dissolved in 0.1 mL of 30% methanol and filtered through 0.2 μm filters (Millex-FG, Millipore, MA, USA). Surrogate analogs, i.e. 100 ng of dihydrojasmonic acid (Sigma-Aldrich, Oakville, Canada), and 100 ng D_6_-salicylic acid (CDN Isotopes, Pointe-Claire, Canada), were spiked prior to extraction in all samples. Procedural blanks were prepared in the same manner. Five leaf samples were spiked with 125 ng of SA and 100 ng JA (Sigma-Aldrich) to estimate the recovery rates of the method.

Levels of hormones in the extracts were quantified using an HPLC Agilent 1290 system coupled to a QTOF Agilent 6545 fitted with a Dual AJS ESI ion source operated in negative ionization mode (Agilent Technologies, Inc., Santa Clara, CA). The HPLC separation was performed with a gradient on a reverse-phase phenyl-hexyl Poroshell 120 column (3.0 × 100 mm, 2.7 μm) from Agilent and a mobile phase of water containing 0.1% formic acid (A) and methanol containing 0.1% formic acid (B). The gradient was as follows: 30% phase B for 1 min, followed by a linear increase of phase B to 100% from minutes 1 to 15 and holding of phase B at 100% for 5 min. The flow rate of the mobile phase was 0.2 mL/min with an injection volume of 10 μL. MS scans (m/z 100–1,100) were completed at a scan rate of 3 spectra/s. Prior to analysis, the QTOF was tuned (mass accuracy below 1 ppm), and mass accuracy was maintained throughout the batch using the continuous infusion of a reference mass mix. Compound identification was based on mass spectra and retention times of pure hormone analytical standards. Target analytes were quantified using a 6-points calibration range (50–1,000 ng/mL) based on the extracted chromatogram for [M-H]^−^ ions. Concentrations were calculated from the relative response vs. the surrogate analogs.

### Statistical analysis

RFB disease progression on soybean was analyzed using ImageJ software version 1.49 (Abràmoff et al., 2004), as previously described on 12 soybean leaves per treatment. Disease severity was calculated as the percent leaf infected and results were compared using Student's *t*-test comparisons with JMP software version 11.0 (SAS Statistics, Cary, NC, U.S.A.). SPAD values for chlorophyll content were compared using Student's *t*-test comparisons with JMP software. Data of qRT-PCR were analyzed using the efficiency calibrated mathematical model (Pfaffl, 2001), where efficiency was calculated for each gene using the method of Zhao and Fernald (2005). Differences in relative transcript abundance and absolute concentration of hormones (*n* = 3) were determined using Student's *t*-test comparisons for statistical significance and biological significance of fold changes ≥1.5 or ≤ −1.5.

### Gene network analysis

Abundance fluxes of soybean-responsive genes exhibiting statistically and biologically significant differences as a result of biochar amendment and following exposure to *R. solani* infections were mapped onto the primary metabolic pathways by reconstruction of data available in the Kyoto Encyclopedia of Genes and Genomes (KEGG) database (http://www.genome.jp/kegg/) and previously published literature.

## Results

### Experiment 1-biochar decreases photosynthesis and increases the severity of RFB in soybean

**Figure 1:**
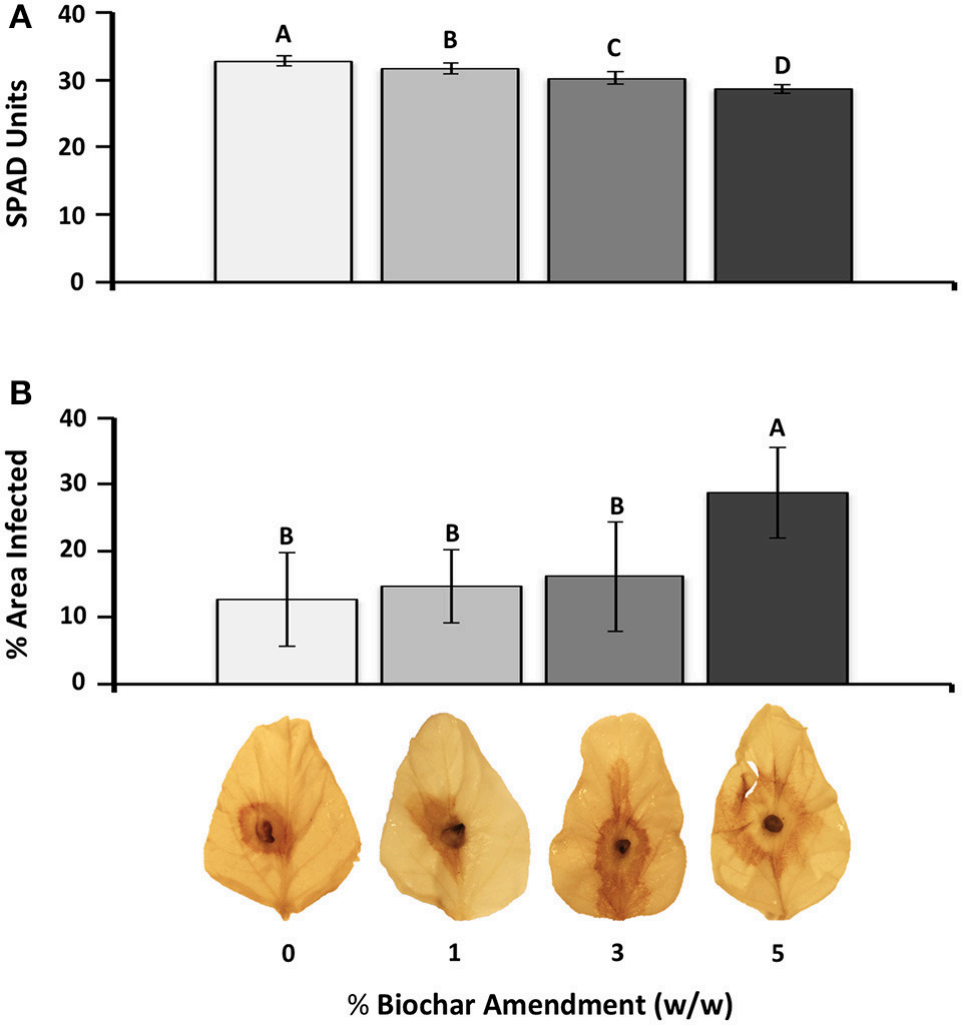
**Effect of biochar amendment rates on soybean chlorophyll content and susceptibility to RFB disease. (A)** Chlorophyll content in response to biochar amendment. Chlorophyll readings were taken on the two fully expanded unifoliate leaves of each plant (*n* = 12) and are represented as SPAD units. **(B)** Average leaf necrosis on detached leaves across different biochar amendment rates. Percent leaf area infected based on the area of necrosis as calculated using Image J software (*n* = 5). Letters represent significant differences based on Student's *t*-test (*P* < 0.05). Bars represent 95% confidence intervals.

Increasing biochar amendment rates significantly (*P* < 0.05) decreased chlorophyll content following 2 weeks of biochar exposure (Figure 1A). Upon infection, the percent leaf area of plants amended with 5% biochar had significantly more necrotic lesions resulting in a 2.26 fold increase in the percent leaf area infected compared with those grown without biochar. At 1 and 3% biochar-amendments, RFB disease severity was not significantly different from that measured in leaves of plants grown in the absence of biochar (0%) (Figure 1B).

**Figure 2:**
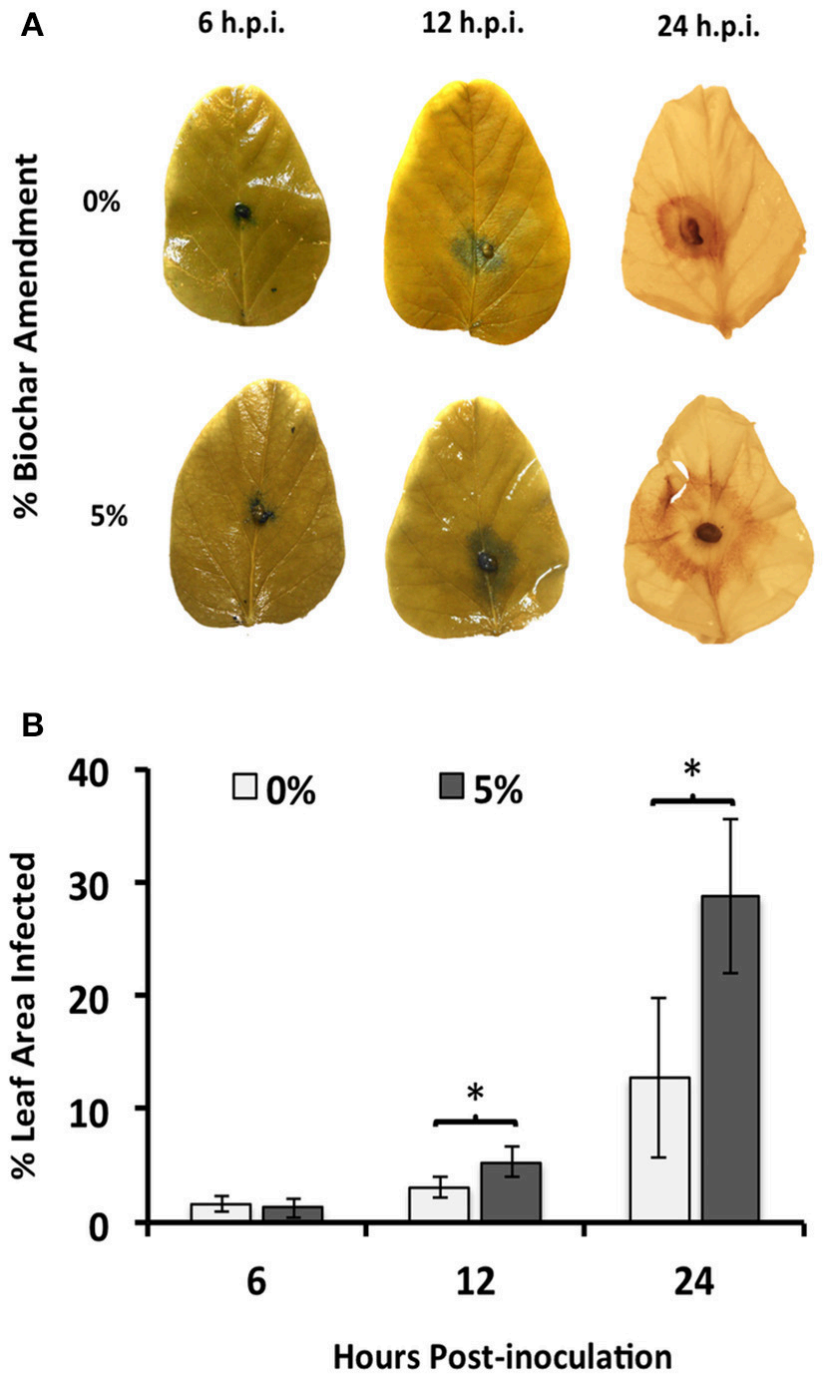
**Time course of *R. solani* infection upon 5% biochar amendment. (A)** Average hyphal expansion at early infection stages (6 and 12 h.p.i.) and necrosis at late infection stages (24 h.p.i.) on detached leaves of soybean plants grown in the presence (5%) or absence (0%) of biochar amendment (w/w). One representative leaf is shown for each time point. **(B)** Average percent leaf area infected on detached leaves of soybean plants grown in the presence (5%) or absence (0%) of biochar amendment (w/w). Percent leaf area infected was determined by calculating the hyphal expansion (6 and 12 h.p.i.) or area of necrosis (24 h.p.i.) using Image J software (*n* = 12). Stars represent statistically significant differences between treatments using Student's *t*-test comparisons (*P* < 0.05), while bars represent 95% confidence intervals.

Leaf area of the soybean plants exposed to 5% biochar and infected with *R. solani* showed that RFB disease progressed rapidly, with significantly (*P* < 0.05) more percent leaf infection (1.68-fold) as early as 12 h.p.i. compared to that of leaves of plants grown in the absence of biochar (Figure 2). No effect of biochar on disease severity was detected 6 h.p.i.

### Experiment 2-fluctuation of soybean gene abundance in response to biochar application

**Figure 3:**
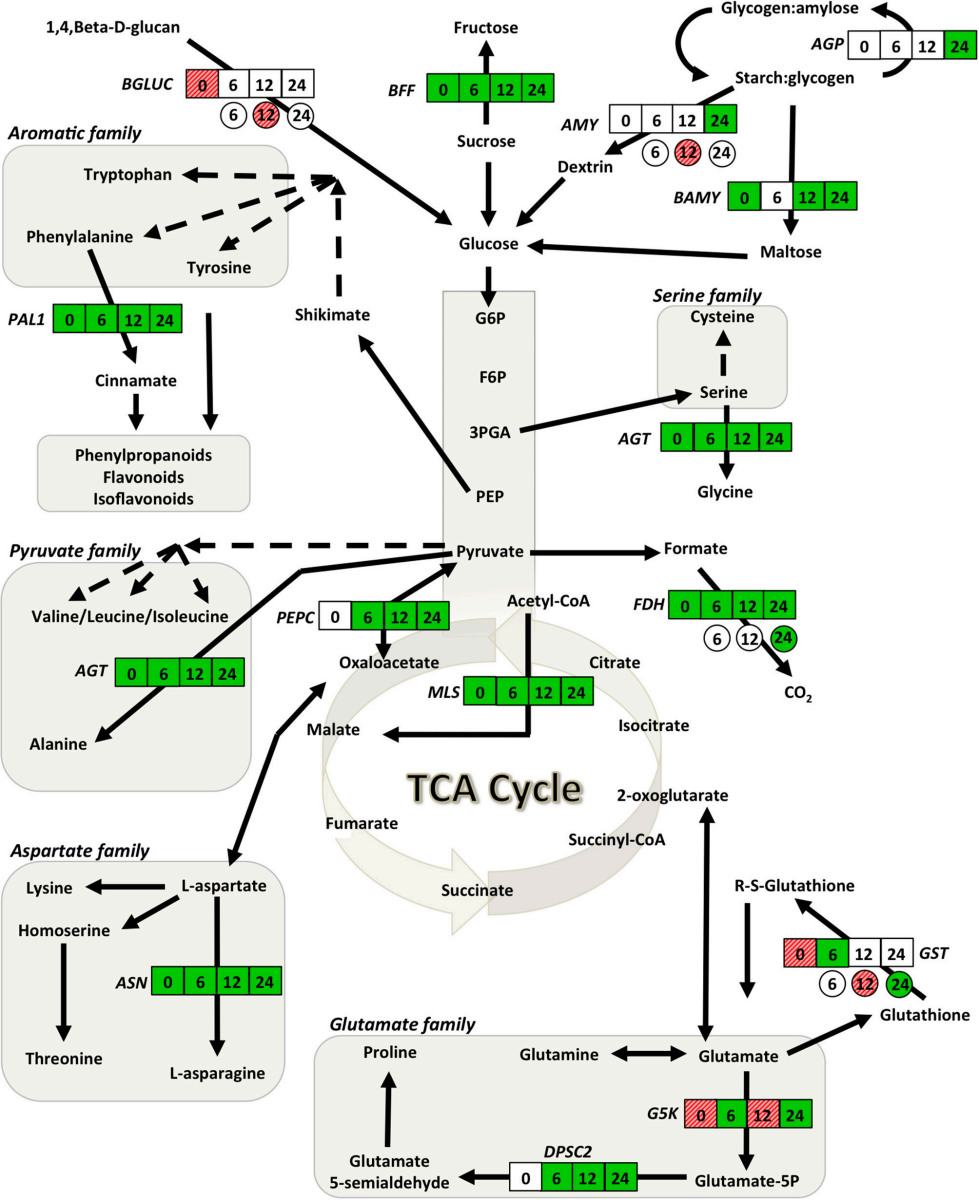
**Gene network analysis showing the time course fluctuations of transcript abundance of soybean and *Rhizoctonia* responsive genes in leaves of plants grown in the presence of 5% (w/w) biochar**. Transcript fold changes of genes associated with primary metabolism for soybean (square) and *R. solani* (circle) were quantified at 0, 6, 12, and 24 h.p.i. Statistically significant (*P* < 0.05) and biologically significant (fold change ≥1.5 or ≤ −1.5) differences in transcript abundances are indicated in boxes/circles where green represents down-regulation, hashed-red up-regulation, and white represents no significant differences (*n* = 3). *AGP*, alpha-glucan phosphorylase; *AGT*, alanine-glyoxylate transaminase; *AMY*, alpha-amylase; *ASN*, asparagine synthetase; *BAMY*, beta-amylase; *BFF*, beta-fructo-furanosidase; *BGLUC*, beta-glucosidase; *DPSC2*, delta-1-pyrroline-5-carboxylate synthase 2; *FDH*, formate dehydrogenase; *G5K*, glutamate-5-kinase; *GST*, glutathione-S-transferase; *MLS*, malate synthase; *PAL*, phenylalanine ammonia lyase 1; *PEPC*, phosphoenolpyruvate carboxykinase 1.

**Figure 4:**
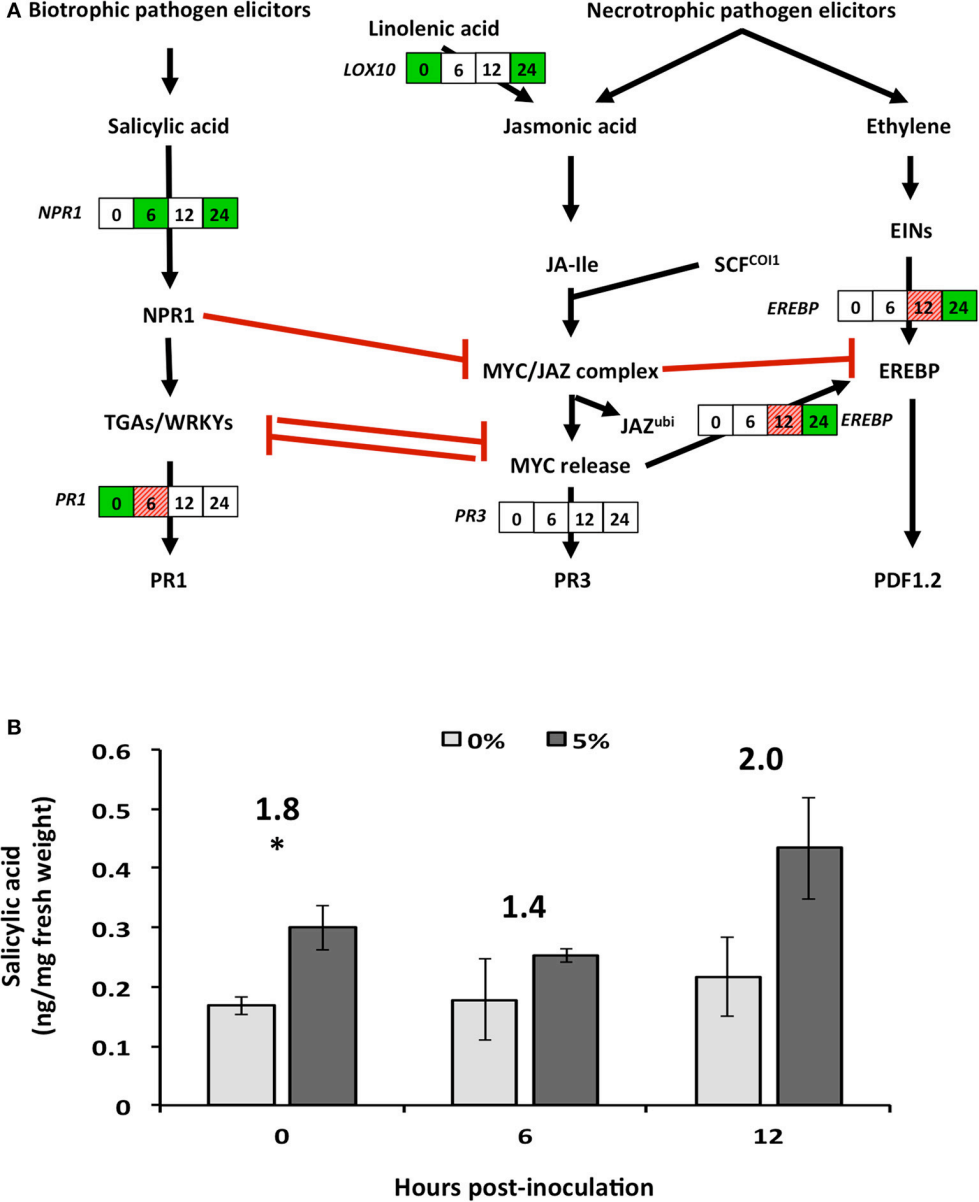
**Effect of biochar amendment on altered soybean genes and plant hormones associated with secondary metabolism**. Soybean plants were grown in the presence (5%) or absence (0%) of biochar amendment (w/w) and transcript relative abundance and salicylic acid levels were quantified at 0, 6, 12, and 24 h.p.i. **(A)** Boxes represent soybean transcript abundance fold changes at 0, 6, 12, and 24 h.p.i. from left to right, respectively. Statistically significant (*P* < 0.05) and biologically significant (fold change ≥1.5 or ≤ −1.5) differences in transcript abundances are indicated in boxes where green represents down-regulation, hashed-red represents up-regulation, and white represents no significant differences (*n* = 3). **(B)** Salicylic acid hormone levels (ng/mg fresh weight) are represented as bar graph in the pathway. Stars represent statistically (*P* < 0.05) and biologically (fold change >1.5) significant differences in SA levels (*n* = 3). Numbers above the bars represent fold changes in hormone levels. *EREBP*, ethylene-responsive element-binding protein 13; *LOX10*, lipoxygenase 10; *NPR1*, non-expresser of PR 1; *PR1*, pathogenesis-related protein 1; *PR3*, pathogenesis-related protein 3.

Compared to plants grown in the absence of biochar, genes involved in glycolysis, the TCA cycle, starch, amino acid and glutathione metabolism together with those associated with plant defense were affected following 5% biochar exposure. Of the 19 genes examined, 9 (*FDH, MLS, AGT, ASN, PAL1, BAMY, BFF PR1, LOX10*) were significantly down-regulated, 3 (*G5K, GST, BGLUC*) were up-regulated and the remaining 7 (*PEPC, DPSC, AGP, AMY, NPR1, PR3, EREBP)* were not affected (Figures 3, 4; Supplementary Table S1). The largest decreases in transcript abundances were associated with the TCA cycle with fold changes of −100 and −8.33 for *MLS* and *FDH*, respectively (Supplementary Table S1). Additionally, transcripts associated with amino acids metabolism (*ASN, AGT*), and starch and carbohydrate metabolism (*BAMY, BFF*) were reduced 4 fold or more (Supplementary Table S1). Large up-regulations were observed in two genes; glutamate-5-kinase (*G5K*) and glutathione-S-transferase (*GST*) with fold changes of 7.33 and 3.00, respectively (Supplementary Table S1).

### Biochar-induced susceptibility of soybean to *R. solani* is partly mediated by down-regulation of soybean genes and changes in salicylic acid levels

In response to biochar and infection by *R. solani*, transcripts involved in the TCA cycle (*FDH, MLS*, and *PEPC*), amino acid metabolism (*AGT, ASN*, DPSC2, *G5K*, and *PAL*1) and glutathione metabolism (*GST*) were down-regulated as early as 6 h.p.i., with fold changes ranging from −3.85 to −1.54 (Supplementary Table S1). Except for *G5K* which was up-regulated at 12 h.p.i., a steady reduction was observed for all of the above genes 12 and 24 h.p.i. (Figure 3; Supplementary Table S1). The largest decreases in transcript abundances were observed 24 h.p.i. for genes associated with the TCA cycle with fold changes of −50.0, −12.5, and −9.09 for *MLS, PEPC*, and *FDH*, respectively (Figure 3; Supplementary Table S1). Genes involved in starch and carbohydrate metabolism were typically down-regulated at 24 h.p.i. (Figure 3; Supplementary Table S1).

The genes associated with plant defense mechanisms involved in SA pathway (*NPR1*) were down-regulated as early as 12 h.p.i., while those involved in the JA (*LOX10 and EREBP*) and phenylpropanoid (*PAL1*) pathways were only down-regulated at later time points (Figures 3, 4A). However, genes down-stream of the transcription factors were typically not significantly altered with the exception of pathogenesis related-protein 1 (*PR1*), which was significantly up-regulated 6 h.p.i. (Figure 4A).

Hormonal analysis of salicylic acid (SA) revealed strong recovery rates of 102 ± 4.6%, while jasmonic acid (JA) levels were typically below the method detection limit, and as such were not analyzed further (Supplementary Table S2). Prior to infection and in the presence of 5% biochar, relative levels of SA in soybean tissues were significantly (*P* < 0.05) and biologically (fold change >1.5) higher compared to levels in tissues of plants grown in the absence of biochar (Figure 4B). On infection with *R. solani*, no changes in SA content were observed in tissues of plants grown with and without biochar at 6 or 12 h.p.i. (Figure 4B). SA levels at 24 h.p.i. could not be analyzed due to lack of SA detection and its surrogate standard D_6_-SA in 0% biochar treatments (Supplementary Table S2). Reasons for this remain unclear, however the presence of signals for H_2_-JA seem to suggest some strong matrix effect in SA analysis occurred at this time point.

### *Rhizoctonia solani* transcripts are altered during soybean infection in response to biochar

**Figure 5:**
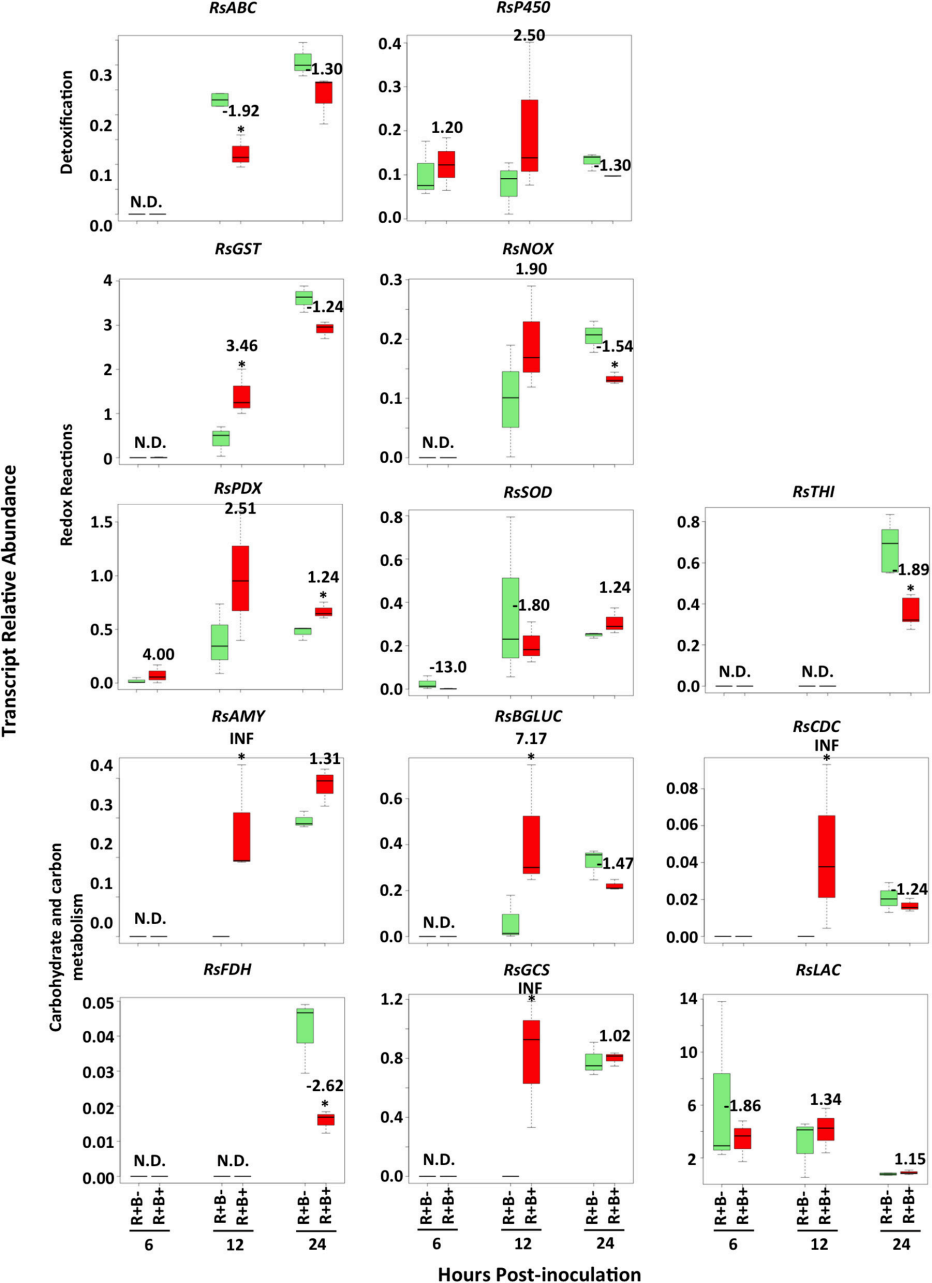
**Time course of *Rhizoctonia solani* transcript abundance changes when infecting leaves of soybean plants that had grown in the presence (B+) or absence (B−) of 5% biochar amendment at 6, 12 and 24 h.p.i**. Stars represent statistically (*P* < 0.05) and biologically (fold change ≥1.5 or ≤ −1.5) significant differences in *R. solani* transcript abundance using Student's *t*-test comparisons (*n* = 3). Numbers represent fold changes, where a positive number represents an increase and a negative number a decrease in transcript abundance in biochar amended treatments compared to controls without biochar amendment. N.D. denotes that the level of transcript was below the detection threshold and was therefore not detected. *RsABC*, ABC transporter; *RsAMY*, alpha-amylase; *RsBGLUC*, beta-glucosidase; *RsCDC*, chitin deacetylase; *RsFDH*, formate dehydrogenase; *RsGCS*, glycogen synthase; *RsGST*, glutathione-s-transferase; *RsLAC*, laccase precursor; *RsNOX*, NADH oxidase; *RsP450*, cytochrome P450 monoxygenase pc-12; *RsPDX*, pyridoxal-repdendant decarboxylase; *RsSOD*, Cu/Zn superoxide dismutase; *RsTHI*, thiamine biosynthesis.

Changes in *R. solani* transcript abundances in response to biochar did not appear before 12 h.p.i. (Figure 5). Abundance of transcripts associated with fungal redox reactions such as NADH oxidase (*RsNOX*) and thiamine biosynthesis (*RsTHI*) were reduced (−1.54 and −1.89, respectively), while that of superoxide dismutase (*RsSOD*) was unaffected (Figure 5; Supplementary Table S3). The *R. solani* ABC transporter (*RsABC*) transcript was down-regulated by −1.92 fold. Five transcripts of *R. solani* were up-regulated: four involved in carbohydrate metabolism [alpha-amylase (*RsAMY*), beta-glucosidase (*RsBGLUC*), glycogen synthase (*RsGCS*) and chitin deacetylase (*RsCDC*)] and glutathione-S-transferase (*RsGST*) were up-regulated with fold changes ranging from 3.46 to infinity (INF) (Figure 5; Supplementary Table S3).

## Discussion

We provide the first evidence that potting mix amended with biochar made from maple bark is conducive to RFB disease leading to increased disease severity. These results are in agreement with our previous study showing that the same type of biochar induced root disease in soybean (Copley et al., 2015b). Our results also provide strong evidence that biochar, which is spatially separated from the pathogen, is linked to the down-regulation of a suite of genes associated with the plant's primary and secondary metabolism, and changes in SA hormonal balance, which in turn caused alterations in *R. solani* transcript abundance. These results are in agreement with the recent study of Viger et al. (2014) that showed down-regulation of a large number of Arabidopsis transcripts related to plant defense with amendment of biochar made from poplar woodchip. In contrast with the data provided here, previous reports showed that incorporation of biochar into potting mix reduced damage caused by foliar and stem pathogens (Elad et al., 2010; Graber et al., 2010; Meller Harel et al., 2012; Zwart and Kim, 2012) and that disease reduction was attributed to biochar-inudced systemic resistance via transcriptional changes of 5 genes linked to plant defense pathways (Meller Harel et al., 2012). It is becoming apparent that the type and concetration of biochar and the conditions which work in one pathosystem may not necessirly work in the same manner in other systems.

In this study, soybean chlorophyll readings decreased with increasing rates of biochar amendment, suggesting that biochar can alter either photosynthetic rates directly or by causing changes in potting substrate pH and electrical conductivity (Copley et al., 2015b) resulting in reduced nutrient availability for the production of photosynthates. Other studies also reported significant reduction in chlorophyll content with biochar (Asai et al., 2009; Kammann et al., 2011). Exposure to biochar did not affect transcript abundance of the soybean storage gene alpha-glucanphosphorylase (*AGP*) and carbohydrate catalytic gene alpha-amylase (*AMY*), but had a drastic effect on gene abundance of beta-amylase (*BAMY*). In Arabidopsis, increased growth in response to biochar amendment had no effect on genes controlling photosynthesis or carbohydrate storage, leading the authors to suggest that the stimulated growth is due to other factors such as increased auxin and brassinosteroid signaling (Viger et al., 2014). Damage caused by foliar pathogens affects supply and translocation of photosynthates (Statler, 1988; Bolton, 2009). For example, *AGP* was significantly down-regulated along with other photosynthesis-associated genes in response to infection (Copley et al., 2015a); however in this study the presence of biochar negates their down-regulation in response to infection. Taken together, these results imply that there is a complex interaction between pathogens and biochar.

Soybean genes involved in amino acid metabolism and the TCA cycle were generally down-regulated, with the exception of glutathione-S-transferase (*GST*) and glutamate-5-kinase (*G5K*), two genes leading to ROS scavenging products (i.e., glutathione and proline, respectively) (Gill and Tuteja, 2010; Szabados and Savouré, 2010). These results agree with those reported by Viger et al. (2014). The increase in *GST* and *G5K* transcript abundances in plants exposed to biochar but not infected indicates that biochar is favorable in creating oxidative stress in plants. Maple bark biochar contains oxalic acid, benzoic acid, octanoic acid and benzaldehyde (Copley et al., 2015b), compounds that are potentially phytotoxic (Takijima, 1964; Ulbright et al., 1982; Kaur and Kaushik, 2005), and conducive to oxidative stress (Liu et al., 2013; Deng et al., 2015; Singh, 2015).

Plant cell walls are loosened by expansins or endo-(1,4)-beta-D-glucanases during growth, or strengthened during times of mechanical stress (Cosgrove, 2005). The up-regulation of the downstream soybean gene *BGLUC* when grown in biochar (i.e., 0 h.p.i.) suggests that cell wall plasticity may have been affected, or that plants have an increased growth rate in response to biochar amendment, although no differences in plant height or mass were observed with biochar amendment at the unifoliate leaf stage (data not shown). The loosening of the cell walls due to increased *BGLUC* expression may have likely facilitated entry points for *R. solani* resulting in earlier and faster colonization. The expression of the plant-derived gene *BGLUC* was similarly expressed during RFB colonization, suggesting that plant cells may have been attempting to reinforce their cell walls, a notion that remains open to speculation. Intriguingly, coupled with soybean *BGLUC* up-regulation prior to infection, there was significant up-regulation of *R. solani* beta-glucosidase (*RsBGLUC*) 12 h.p.i indicating that more monomeric sugars are freely available to the pathogen when infecting plants previously grown in biochar.

During plant-pathogen interactions, energy, nitrogen and carbon sources are known to shift toward secondary metabolite producing pathways (Bolton, 2009). The general down-regulation of genes involved in the TCA cycle, amino acid metabolism and carbohydrate metabolism is indicative of down-regulation of downstream secondary metabolic pathways (Bolton, 2009; Conrath, 2011). Indeed, phenylalanine ammonia lyase 1 (*PAL*), a gene associated with secondary metabolism, together with lipoxygenase (*LOX10*), and pathogenesis-related (PR) proteins (*PR1*), were down-regulated in response to biochar amendment in the absence of *R. solani* infection. These results agree with those reported by Viger et al. (2014), in which similar levels of biochar amendment caused down-regulation of *LOX* and the Arabidopsis ethylene response factor (*ERF15*). In this study, SA levels significantly increased in response to biochar amendment (0 h.p.i.), but did not change upon infection. However, biochar potentiated the early expression of the SA inducible marker gene *PR1* at 6 h.p.i., which acts downstream of the SA biosynthetic pathway. Although no plant cultivars are fully resistant to *R. solani*, some crop species, such as rice, have varieties with increased tolerance to *R. solani* due to activation of JA, *LOX*, and *PAL* (Jayaraj et al., 2010; Taheri and Tarighi, 2010) and not SA. The intricate cross-talk between SA and JA pathways is only partly understood, although increases in SA typically lead to decreases in JA via multiple mechanisms (Caarls et al., 2015). The activation of SA and its down-stream genes observed in this study suggests that maple bark biochar may in fact prime SA and not JA resulting in susceptibility to *R. solani* suggesting that maple bark biochar is insufficient for priming soybean defenses against *R. solani*.

Interestingly, the decreases in soybean transcripts involved in secondary metabolism and defense corresponded to decreases in *R. solani* transcript abundances associated with detoxification and with cell wall restructuring. Fungal ABC transporters act as efflux pumps exporting toxic compounds out of fungal cells and reducing concentrations of antibiotics and toxic compounds (Duffy et al., 2003). The decrease in the abundance of ABC transporters during infection of soybean leaves from plants grown in biochar strengthen the assumption that toxic compounds (i.e., antibiotic or anti-deterrent proteins and/or metabolites) released by the plant were not produced in sufficient amounts compared to those grown in the absence of biochar. Cytochrome P450 genes are another cluster of genes that play an important role in fungal metabolism of xenobiotics, detoxification, and secondary metabolite production (Guengerich, 2001; Bhatnagar et al., 2003; Mukherjee and Kenerley, 2010). These genes were highly up-regulated in *R. solani* in response to biotic stress (Chamoun and Jabaji, 2011; Gkarmiri et al., 2015); however, no change in *R. solani* cytochrome P450 during interaction with soybean exposed to biochar was detected suggesting constitutive expression during plant invasion.

Further support for a decrease in soybean secondary metabolism is the increase in *R. solani* chitin deacetylase (*RsCDC*), a gene involved in converting hyphal chitin to chitosoan. Decreases in *RsCDC* transcript abundance were associated with hardening of cell walls during confrontation with antagonistic bacteria such as *Serratia* species (Gkarmiri et al., 2015). The down-regulation of this gene during infection of soybean exposed to biochar suggests an increased growth rate of *R. solani*, and a lesser need for it to maintain thicker cell walls for defense against soybean secondary metabolites.

Reactive oxygen species (ROS) play an important role in host attack, though the pathogen must be capable of defending itself against its own ROS as well as the host ROS. A wide array of ROS quenching mechanisms exists and has been shown to be essential for successful host invasion (Shetty et al., 2008). Several studies have reported on the varied gene expression of *R. solani* ROS quenching genes under different types of stress. For example, Foley et al. (2016) reported up-regulation of *R. solani* Cu/Zn superoxide dismutase (*RsSOD*), but not NAD(P)H oxidase (*RsNOX*) during infection of wheat, while Gkarmiri et al. (2015), Samsatly et al. (2015) and Chamoun and Jabaji (2011) saw up-regulation of vitamin B6 related genes during abiotic and biotic stresses. In this study, varying effects were observed for *R. solani* genes involved in redox reactions emphasizing their alternative roles in defense and attack. *R. solani* glutathione-S-transferase (*RsGST)* and pyridoxal-dependant decarboxylase (*RsPDX*) had stronger roles during early and late stages of infection of leaves from soybean grown in biochar, respectively. *RsNOX* and thiamine synthase (*RsTHI*) were down-regulated during infection of soybean leaves grown in biochar suggesting that they may have more important roles in defense against soybean secondary metabolites, whose transcripts were higher in soybean leaves grown in the absence of biochar.

After successful invasion and evasion of host defense mechanisms, it is important for the pathogen to successfully utilize its host's energy resources. The *R. solani* carbohydrate degrading transcripts alpha-amylase (*RsAMY*) and beta-glucosidase (*RsBGLUC*) increased in abundance when *R. solani* was infecting leaves from soybean plants grown in biochar compared to those not grown in biochar. These increases occurred in parallel to increases in transcript abundance of the *R. solani* carbohydrate storage gene, glycogen synthase (*RsGCS*), further supporting the idea that carbohydrates were more readily available to the pathogen from leaves of soybean plants grown in biochar amended potting mixtures. The lack of change in transcript abundance of the laccase (*RsLAC*) gene further supports the idea that sufficient carbohydrates were available for *R. solani* infecting soybean plants grown in 5% biochar amended potting mixtures. This is based on the evidence that laccase genes are typically only up-regulated when carbohydrates are not readily available and lignin degradation becomes necessary, or during high phenolic stress (De Souza, 2013). Taken together, these results suggest that sufficient amounts of carbohydrates were available to *R. solani* despite decreased soybean chlorophyll content, and that *R. solani* was exposed to reduced amounts of secondary metabolites when infecting plants grown in biochar.

In summary, this is the first study to report that plant exposure to biochar results not only in alterations of disease severity, but also indirectly affects the pathogen transcript abundance by modulation of plant gene expression and salicylic acid levels, emphasizing that biochar-plant-pathogen interactions are complex. The need to examine the molecular responses of plants to different types of biochar at a broader scale in an attempt to link, and possibly predict, the effect of biochar on plant growth and defense mechanisms merits in-depth investigations.

## Author contributions

Conception and design of the study: TC and SJ. Acquisition of data for the study: TC and SB. Analysis of data for the work: TC and SB. Interpretation of data for the work: TC and SJ. Manuscript revision and approval: TC, SB, and SJ.

## Funding

Financial support was provided as a Discovery grant to SJ by the National Sciences and Engineering Research Council of Canada (RGPIN137135-201 and RGPIN-2016-04805) and partially supported by the academic startup grant of SB. We thank P. Ceresini for his technical advise on incoulation experiments of detached soybean leaves. The authors would like to thank Agilent Technologies for their technical support and access to equipment.

### Conflict of interest statement

The authors declare that the research was conducted in the absence of any commercial or financial relationships that could be construed as a potential conflict of interest.
