# Supplementary material for: Biochar Amendment Modifies Expression of Soybean and Rhizoctonia solani Genes Leading to Increased Severity of Rhizoctonia Foliar Blight
Source: Front Plant Sci. 2017 Feb 21;8:221. doi: 10.3389/fpls.2017.00221 (PMC5318381; doi:10.3389/fpls.2017.00221)
Supplement: Supplementary file 1 [file Table1.docx]

**Supplementary Table S1.** Effect of biochar on abundance of soybean transcripts associated with primary metabolism during interaction with *Rhizoctonia solani*

|  |  | **Hours post-inoculation (h.p.i)** | | | | | | | | | | |
| --- | --- | --- | --- | --- | --- | --- | --- | --- | --- | --- | --- | --- |
|  |  | **6** | | |  | **12** | | |  | **24** | | |
| **Biochar Treatment^#^** | | **R+B-** | **R+B+** | **Fold Change** |  | **R+B-** | **R+B+** | **Fold Change** |  | **R+B-** | **R+B+** | **Fold Change^%^** |
| **TCA cycle** | **Formate dehydrogenase (*FDH*)** | 0.13 A | 0.07 B | **-1.85** |  | 1.58 A | 0.30 B | **-5.26** |  | 1.78 A | 0.19 B | **-9.09** |
|  | **Malate synthase (*MLS*)** | 0.41 A | 0.21 B | **-1.96** |  | 37.52 A | 0.87 B | **-50.0** |  | 168.0 A | 3.35 B | **-50.0** |
|  | **Phosphenolpyruvate carboxykianse 1 (*PEPC*)** | 0.34* A | 0.22* B | **-1.54** |  | 0.006 A | 0.003 A | -2.00 |  | 0.85 A | 0.07 B | **-12.5** |
| **Amino acid metabolism** | **Alanine-glyoxylate transaminase (*AGT*)** | 0.61 A | 0.52 A | -1.18 |  | 2.81 A | 0.73 B | **-3.85** |  | 7.78 A | 1.19 B | **-6.67** |
|  | **Asparagine synthetase (*ASN*)** | 0.19 A | 0.05 B | **-3.85** |  | 0.11 A | 0.02 B | **-5.56** |  | 0.74 A | 0.27 B | **-2.78** |
|  | **Delta 1-pyrroline-5-carboxylate synthase 2 (*DPSC2*)** | 0.14 A | 0.04 B | **-3.57** |  | 0.34 A | 0.10 A | -3.57 |  | 5.92 A | 0.82 B | **-7.14** |
|  | **Glutamate-5-kinase (*G5K*)** | 0.55 A | 0.25 B | **-2.22** |  | 0.41 B | 1.03 A | **2.51** |  | 5.69 A | 1.91 B | **-2.94** |
|  | **Phenylalanine ammonia lyase 1 (*PAL1*)** | 0.54 A | 0.29 B | **-1.85** |  | 2.85 A | 0.91 B | **-3.12** |  | 4.57 A | 0.54 B | **-8.33** |
| **Glutathione metabolism** | **Glutathione-S-transferase (*GST*)** | 0.12 A | 0.06 B | **-2.00** |  | 0.17 A | 0.30 A | 1.76 |  | 0.90 A | 0.82 A | -1.10 |
| **Starch metabolism** | **Alpha-glucan phosphorylase (*AGP*)** | 8.63 A | 5.92 B | -1.45 |  | 3.46 A | 4.36 A | 1.26 |  | 3.13 A | 0.74 B | **-4.17** |
|  | **Alpha-amylase (*AMY*)** | 0.38 A | 0.67 A | 1.76 |  | 2.57 A | 2.14 A | -1.18 |  | 3.58 A | 1.93 B | **-1.85** |
|  | **Beta-amylase (*BAMY*)** | 14.76 A | 12.16 A | -1.22 |  | 56.65 A | 13.55 B | **-4.17** |  | 18.47 A | 4.58 B | **-4.00** |
| **Carbohydrate metabolism** | **Beta-fructo- furanosidase or invertase (*BFF*)** | 1.37 A | 1.50 A | 1.09 |  | 6.33 A | 1.21 B | **-5.26** |  | 12.19 A | 2.61 B | **-4.76** |
|  | **Beta-glucosidase (*BGLUC*)** | 9.19* A | 5.25* A | -1.75 |  | 2.61* A | 23.40* A | 8.96 |  | 0.13 A | 0.06 A | -2.17 |
| **Secondary metabolism** | **Non-expressor of PR 1 (*NPR1*)** | 2.46 A | 2.14 B | -1.15 |  | 2.68 A | 3.15 A | 1.17 |  | 3.12 A | 2.39 B | -1.32 |
|  | **Pathogenesis-related protein 1 (*PR1*)** | 3.47 B | 6.84 A | **1.97** |  | 7.43 A | 6.34 A | -1.18 |  | 2.32 A | 2.03 A | -1.14 |
|  | **Pathogenesis-related protein 3 (*PR3*)** | 0.13 A | 0.09 B | -1.37 |  | 0.24 A | 0.24 A | -1.01 |  | 1.49 A | 1.50 A | 1.01 |
|  | **Ethylene-responsive element-binding protein 13 (*EREBP*)** | 1.01A | 0.79 B | -1.28 |  | 1.41 B | 2.18 A | **1.54** |  | 1.14 A | 0.68 B | **-1.67** |
|  | **Lipoxygenase 10 (*LOX10*)** | 1.28 A | 0.96 A | -1.32 |  | 2.63 A | 2.12 B | -1.25 |  | 0.51 A | 0.36 B | -1.43 |

Letters represent significant differences at each time point using Student’s t test (*P*<0.05). Fold changes represent the fold change in R+B+ versus R+B- treatments: negative values represent a decrease in transcript relative abundance upon biochar amendment, while positive values represent an increase in transcript relative abundance.

^#^Biochar treatment refers to the absence (B-) or presence (B+) of biochar in the potting substrate. Biochar was amended at a rate of 5% w/w in B+ treatments.

^%^Fold changes in bold represent significant transcript changes based on statistical significance (*P* <0.05) and biological significant (fold change ≥1.5 or ≤-1.5).

*Relative abundances are numbers multiplied by 10^-4^
