# Supplementary material for: Biochar Amendment Modifies Expression of Soybean and Rhizoctonia solani Genes Leading to Increased Severity of Rhizoctonia Foliar Blight
Source: Front Plant Sci. 2017 Feb 21;8:221. doi: 10.3389/fpls.2017.00221 (PMC5318381; doi:10.3389/fpls.2017.00221)
Supplement: Supplementary file 3 [file Table3.docx]

**Supplementary Table S3.** *Rhizoctonia solani* responsive transcript abundance changes in response to biochar during the interaction with soybean

|  |  | **Hours post-inoculation (h.p.i.)** | | | | | | | | | | |
| --- | --- | --- | --- | --- | --- | --- | --- | --- | --- | --- | --- | --- |
|  |  | **6** | | |  | **12** | | |  | **24** | | |
|  | **Biochar Treatment^#^** | **R+B-** | **R+B+** | **Fold Change** |  | **R+B-** | **R+B+** | **Fold Change** |  | **R+B-** | **R+B+** | **Fold Change^%^** |
| **Detoxification** | **ABC transporter (*RsABC*)** | N.D.^$^ | N.D. | N.A.^+^ |  | 0.23 A | 0.12 B | **-1.92** |  | 0.31 A | 0.24 A | -1.30 |
|  | **Cytochrome P450 monooxygenase pc-12 (*RsP450*)** | 0.10 A | 0.12 A | 1.20 |  | 0.08 A | 0.20 A | 2.50 |  | 0.13 A | 0.10 B | -1.30 |
| **Redox reactions** | **Glutathione-S-transferase (*RsGST*)** | N.D. | N.D. | N.A. |  | 0.41 B | 1.42 A | **3.46** |  | 3.60 A | 2.91B | -1.24 |
|  | **NADH oxidase (*RsNOX*)** | N.D. | N.D. | N.A. |  | 0.10 A | 0.19 A | 1.90 |  | 0.20 A | 0.13 B | **-1.54** |
|  | **Pyridoxal-dependent decarboxylase (*RsPDX*)** | 0.02 A | 0.08 B | 4.00 |  | 0.39 A | 0.98 A | 2.51 |  | 0.47 B | 0.67 A | 1.42 |
|  | **Cu/Zn superoxide dismutase (*RsSOD*)** | 0.26* A | 0.02* A | -13.0 |  | 0.36 A | 0.20 A | -1.80 |  | 0.25 A | 0.31 A | -1.24 |
|  | **Thiamine biosynthesis (*RsTHI*)** | N.D. | N.D. | N.A. |  | N.D. | N.D. | N.A. |  | 0.68 A | 0.36 B | **-1.89** |
| **Carbohydrate and carbon metabolism** | **Alpha-amylase (*RsAMY*)** | N.D. | N.D. | N.A. |  | N.D. B | 0.27 A | **INF^&^** |  | 0.29 A | 0.38 A | 1.31 |
|  | **Beta-glucosidase (*RsBGLUC*)** | N.D. | N.D. | N.A. |  | 0.06 B | 0.43 A | **7.17** |  | 0.32 A | 0.22 A | -1.45 |
|  | **Chitin deacetylase (*RsCDC*)** | N.D. | N.D. | N.A. |  | N.D. B | 0.04 A | **INF** |  | 0.21* A | 0.17* B | -1.24 |
|  | **Formate dehydrogenase (*RsFDH*)** | N.D. | N.D. | N.A. |  | N.D. | N.D. | N.A. |  | 0.42* A | 0.16* A | -2.62 |
|  | **Glycogen synthase (*RsGCS*)** | N.D. | N.D. | N.A. |  | N.D. B | 0.81 A | **INF** |  | 0.78 A | 0.80 A | 1.02 |
|  | **Laccase precursor (*RsLAC*)** | 6.34 A | 3.40 A | -1.86 |  | 3.08 A | 4.13 A | 1.34 |  | 0.78 A | 0.90 A | 1.15 |

Letters represent significant differences at each time point using Student’s t test (*P*<0.05). Fold changes represent the fold change in R+B+ versus R+B- treatments: negative values represent a decrease in transcript relative abundance upon biochar amendment, while positive values represent an increase in transcript relative abundance.

^#^Biochar treatment refers to the absence (B-) or presence (B+) of biochar in the potting substrate. Biochar was amended at a rate of 5% w/w in B+ treatments.

^%^ Fold changes in bold represent significant transcript changes based on statistical significance (*P* <0.05) and biological significance (fold change ≥1.5 or ≤-1.5).

^$^ N.D., not detected, C_T_ value below the threshold limit

^+^ N.A., not applicable as C_T_ values for both treatments were below the threshold limit

* Relative abundances are numbers multiplied by 10^-1^

^&^ INF, infinite fold increase due to levels in one treatment being below the detection threshold
